# Supplementary figures and images for: Regulation of sedimentation rate shapes the evolution of multicellularity in a close unicellular relative of animals
Source: PLoS Biol. 2022 Mar 29;20(3):e3001551. doi: 10.1371/journal.pbio.3001551 (PMC8963540; doi:10.1371/journal.pbio.3001551)

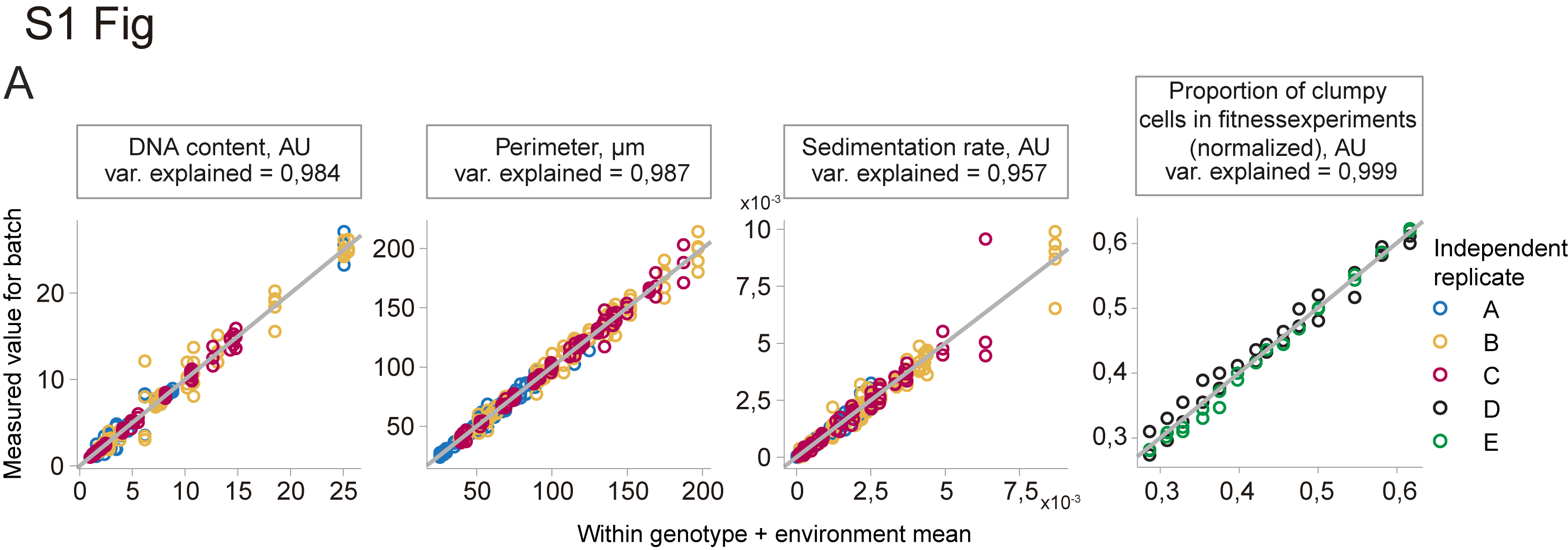

Supplement: S1 Fig — (A) Measurements of technical error or heritability of traits show that the phenotypes measured for the evolved isolates were highly heritable. Arithmetic mean values for 4 key phenotypes were calculated for each genotype + environment combination (horizontal X axis) and plotted against individual measured values (vertical Y axis) across replicates performed on separate days (“batches”—colors). Environments included different temperatures and different timings after cell cycle synchronization. Horizontal facet strips indicate the phenotype, its unit of measure, and the heritability of the trait, i.e., the variance explained by the mean genotype + environment value. Gray lines represent where X = Y. (TIF) [file pbio.3001551.s005.tif]

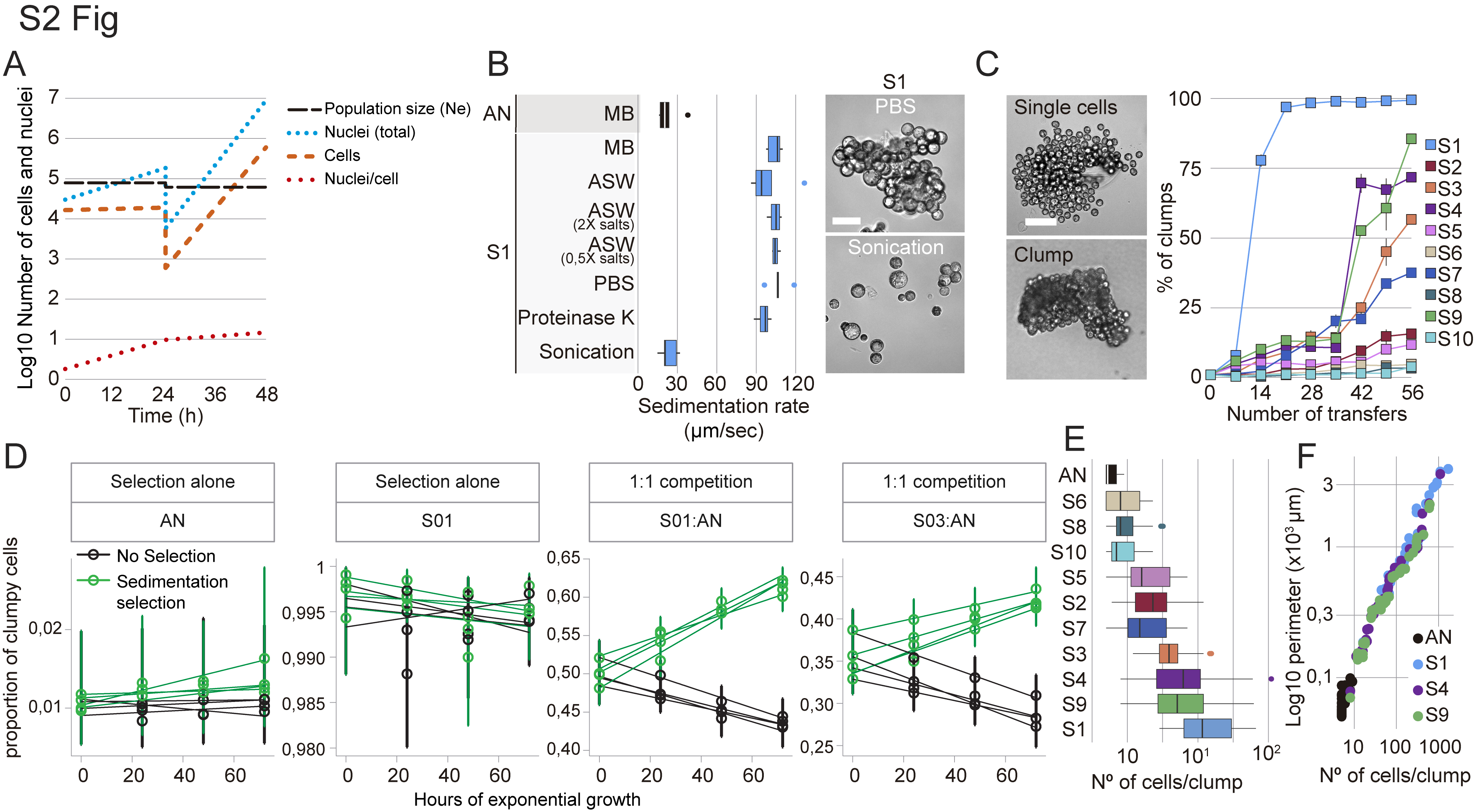

Supplement: S2 Fig — (A) Approximation of the constant population size derived from nuclear doublings over the course of 48 hours. Nuclei numbers were estimated from total cell number and average number of nuclei per cells (after cell sorting). At 24 hours, a constant fraction of cells has been transferred to fresh medium, which induces a bottleneck. The data underlying this figure may be found in S1 Table. (B) Sedimentation rates of S1 clumps after incubation for 2 hours in different media or after sonication. MB = marine broth, ASW = artificial sea water (Salt concentration = 36.4 g/L), 2X (Salts concentration = 72.8 g/L), 0.5X (Salts concentration = 18.2 g/L), PBS = phosphate-buffered saline 1X, Proteinase K 200 μg/mL and sonication (3 pulses of 15 seconds, 10% amplitude). Representative images on the right show the dissociation of S1 clumps after sonication. Bar, 50 μm. (C) Percentage of clumps formed after 72 hours of all evolved mutants per number of transfers (n > 200 coenocytes at cell release per strain). Example images on the left of detached or clumpy cells after cell release. Bar, 50 μm. (D) Raw data for the frequency with respect to time of each independent replicate of strains ancestor (AN) and clumpy evolved isolates S01 and S03 in head-to-head competitions with the ancestral strain (2 days, 2 replicates per day). Each point represents a measurement of a given replicate at the given time point under the 2 selection regimes (colors—Green = in presence of sedimentation rate selection; Black = in absence of selection), and error bars are 95% confidence intervals. Lines are least-squares regressions of each replicate across time points to guide the eye. The data underlying this graph may be found in S4 data; see Methods. (E) Number of cells per clump in all evolved mutants illustrates the linear correlation between sedimentation rate and number of cells per clump. (n = 13 clumps for AN and >50 clumps for all evolved mutants of at least 5 attached cells together). (F) Linear c [file pbio.3001551.s006.tif]

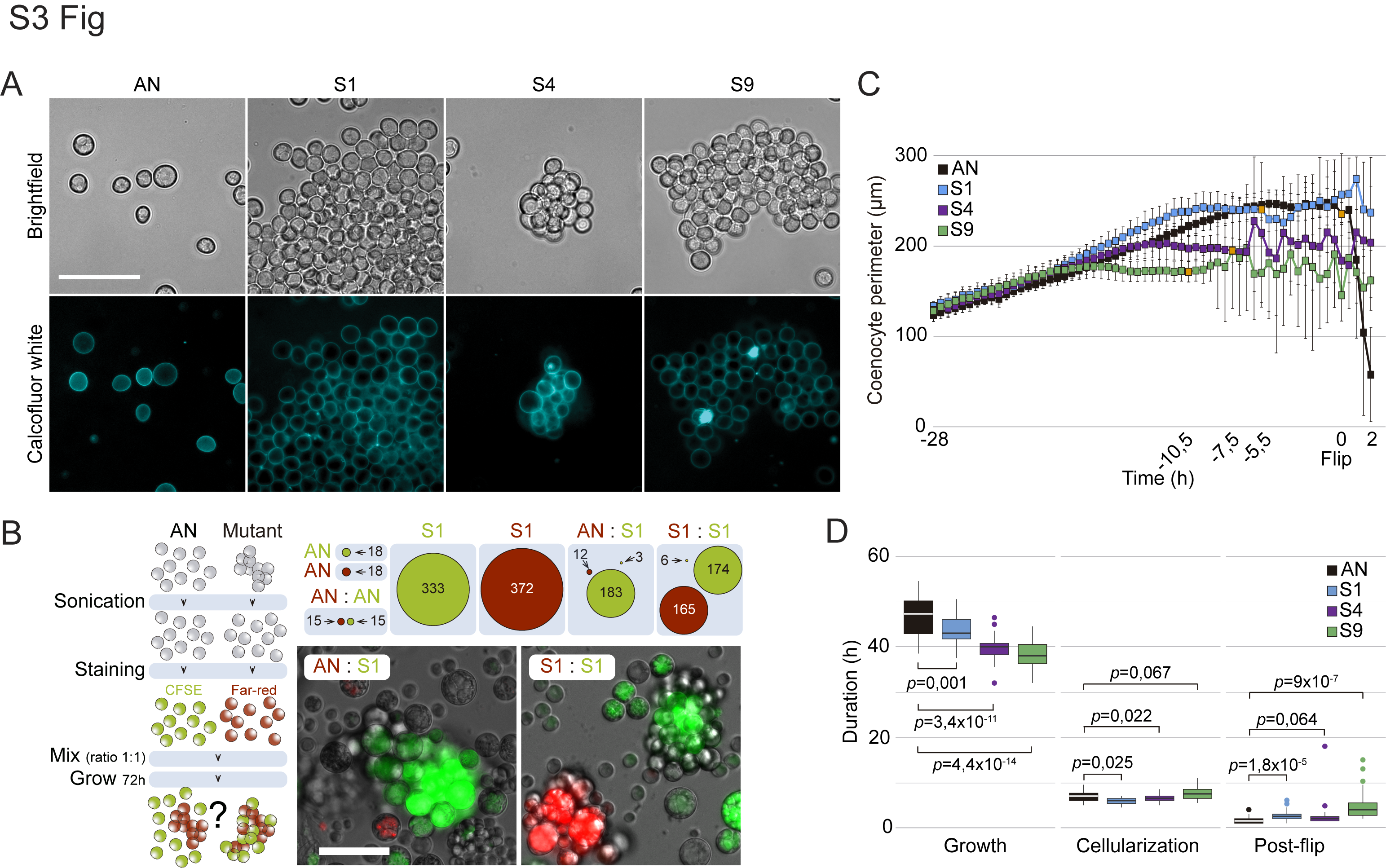

Supplement: S3 Fig — (A) Cell wall staining show presence of a separating cell wall between individual cells in the clumps of all fast-settling mutants. Bar, 50 μm. (B) Experimental design and measurements of S1 clump formation by aggregation. AN and S1 cells are separated by sonication and stained with different cellular dyes before being mixed together for a complete life cycle of 72 hours. Clumps of each separate or mixed colors are then counted by microscopy. Circles represent the number of clumps observed in each condition (more than 5 cells attached together). We show that when S1 is mixed with either AN or itself (1:1 ratio), it mostly forms monocolored clumps. Representative images of monocolored clumps. Bar, 50 μm. (C) Mean coenocyte perimeter over time (10 cell traces per strain) at 12°C, aligned to time 0, reveals discreet differences in coenocyte perimeter and life cell stages among fast-settling mutants. Orange squares represents the flip time point in each trace. (D) Duration of growth, cellularization, and post-flip represented as box-plots at 12°C (n > 28 coenocytes each). The data underlying this figure may be found at https://doi.org/10.6084/m9.figshare.18319232.v1. (TIF) [file pbio.3001551.s007.tif]

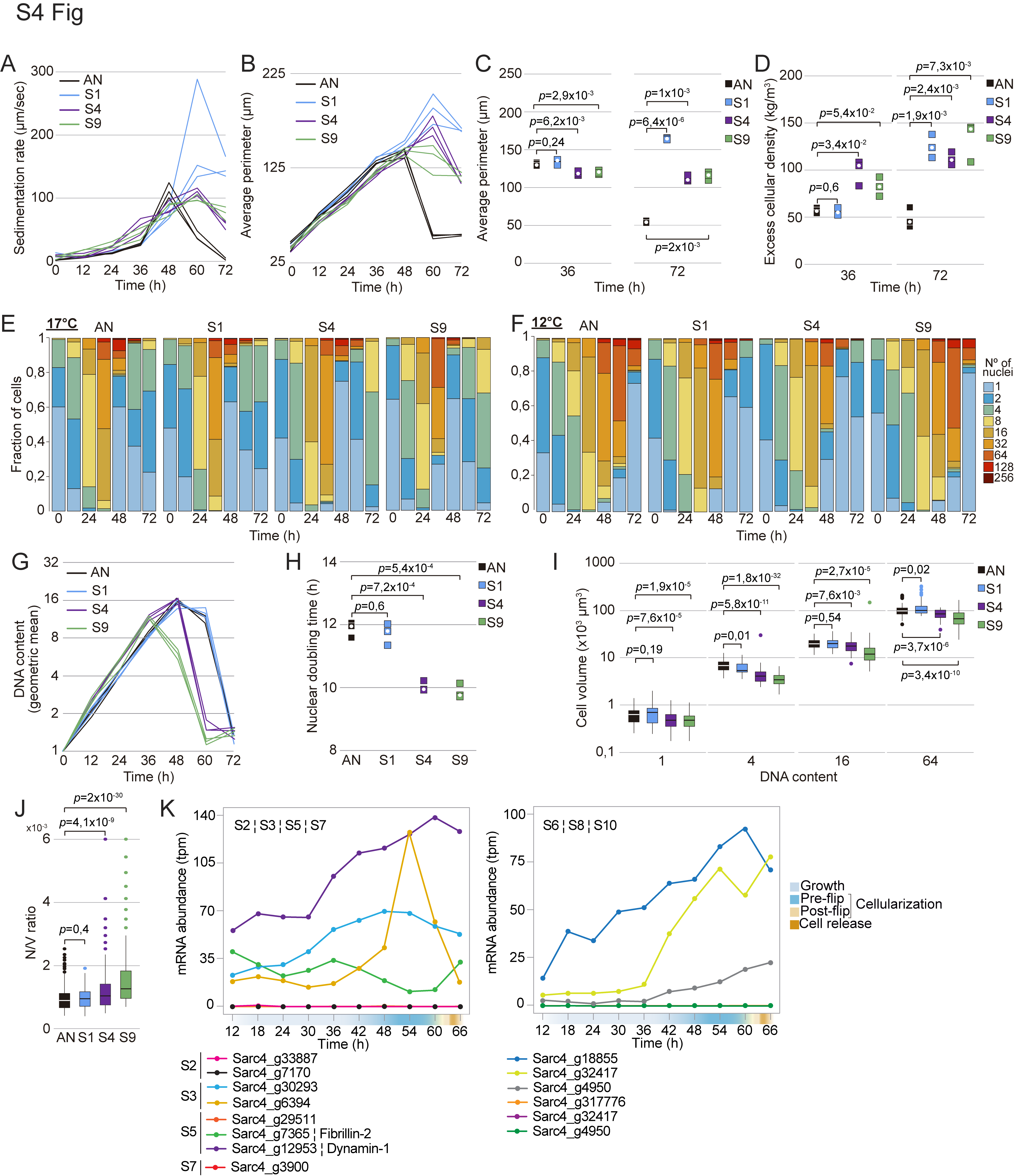

Supplement: S4 Fig — (A) Sedimentation rates of S. arctica AN and evolved mutants during the life cycle at 12°C. Every trace represents an independent experiment. (B) Average perimeter measured from fixed cells every 12 hours over a complete life cycle of 72 hours at 12°C shows that fast-settling mutant increase their size upon cell release. Every trace represents an independent experiment (n > 130 measurements per time point for each independent experiment). (C) Average perimeter of fast-settling cells and clumps at 36 and 72 hours, respectively, show that S4 and S9 single cells and clumps have a smaller size when compared to S1 at 12°C. Every square represents an independent experiment, and the white circle represents the median. (D) Excess cellular density of fast-settling mutants (before cellularization) and clumps (after cellularization) at 36 and 72 hours, respectively. Every square represents an independent experiment, and the white circle represents the median. (E) Distributions of nuclear content of S. arctica AN and fast-settling mutants during the life cycle at 17°C measured by microscopy of DAPI-fixed cells (n > 400 coenocytes per time point). (F) Distributions of nuclear content of S. arctica AN and fast-settling mutants during the life cycle at 12°C measured by microscopy of DAPI-fixed cells (n > 420 coenocytes per time point for each independent experiment). (G) Quantification of mean DNA content per time point for fast-settling mutants grown in marine broth at 12°C. Every trace represents an independent experiment (n > 420 coenocytes per time point for each independent experiment). (H) Nuclear doubling time, calculated by linear regression of mean nuclear content at time points from 0 to 24 hours at 12°C. Every square represents an independent experiment, and the white circle represents the median (n > 420 coenocytes per time point for each independent experiment). (I) Boxplots of cell volume measurements of DAPI-stained fixed cells at 12°C. For 1-, 4-, 16-, and 64-nucle [file pbio.3001551.s008.tif]

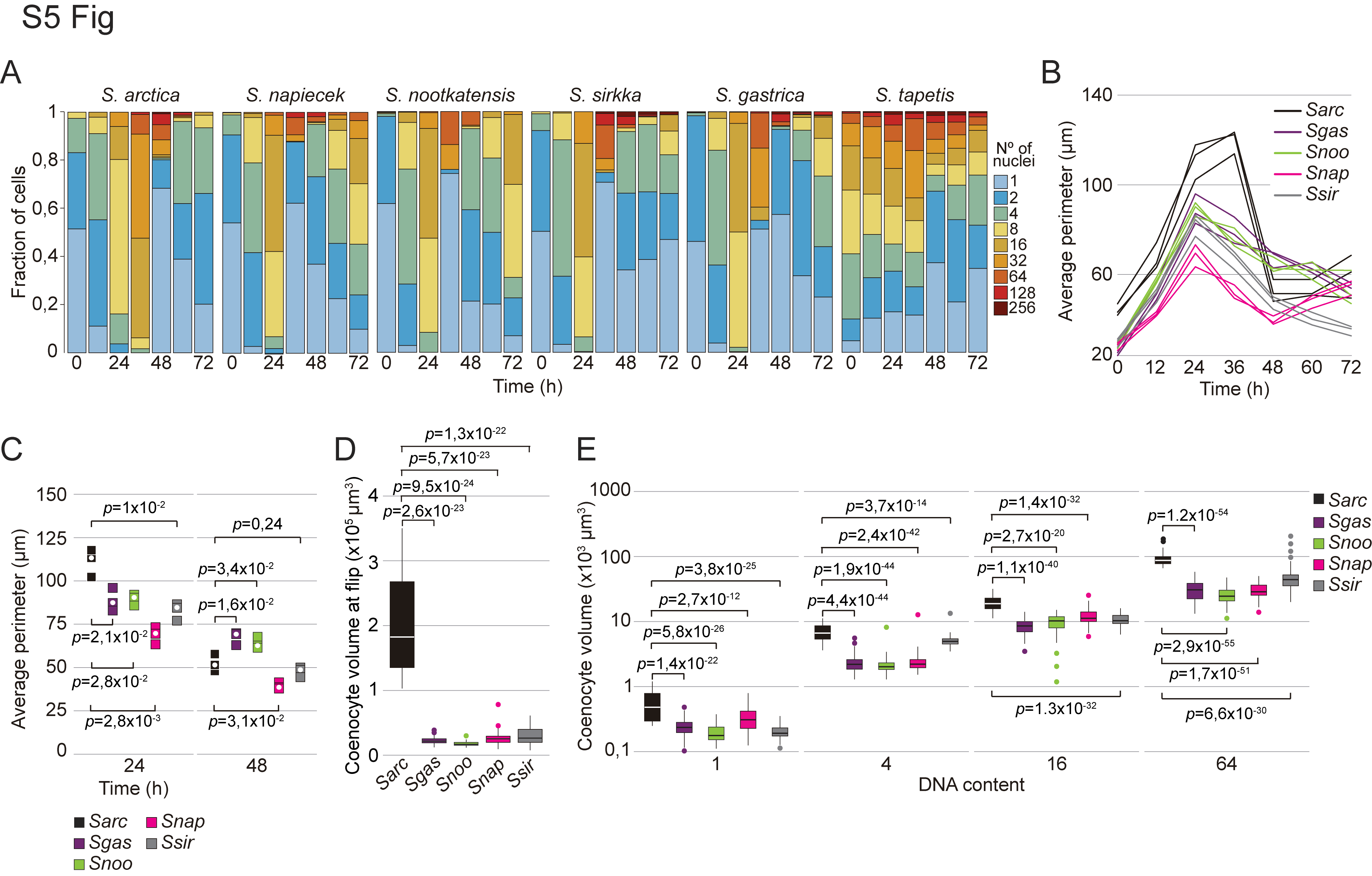

Supplement: S5 Fig — (A) Distributions of nuclear content of Sphaeroforma species during the life cycle at 17°C of DAPI-fixed cells measured by microscopy. Note that S. tapetis is asynchronous compared to all other species (n > 300 coenocytes per time point for each independent experiment). (B) Average perimeter measured from fixed cells every 12 hours over a complete life cycle of 72 hours at 17°C of Sphaeroforma sp. Every trace represents an independent experiment (n > 140 measurements per time point for each independent experiment). (C) Average perimeter of Sphaeroforma sp. cells and clumps at 0, 24, and 48 hours, respectively, show that S. gastrica, S. nootakensis, S. napiecek, and S. sirkka are smaller in size at 17°C. Every square represents an independent experiment, and the white circle represents the median (n > 140 measurements per time point for each independent experiment). (D) Coenocyte volume at flip, measured from time-lapse movies, show that all Sphaeroforma species apart from S. arctica have significantly smaller coenocyte volume at flip (n > 50 coenocytes per strain). (E) Boxplots of cell volume measurements of DAPI-stained fixed Sphaeroforma sp. coenocytes at 17°C. For 1-, 4-, 16-, and 64-nuclei cells. Cells with 1 nucleus represent newborn cells at the end of the experiment. (n > 50 coenocytes per DNA content). The data underlying this figure may be found at https://doi.org/10.6084/m9.figshare.18319232.v1. (TIF) [file pbio.3001551.s009.tif]
