# Supplementary material for: Regulation of sedimentation rate shapes the evolution of multicellularity in a close unicellular relative of animals
Source: PLoS Biol. 2022 Mar 29;20(3):e3001551. doi: 10.1371/journal.pbio.3001551 (PMC8963540; doi:10.1371/journal.pbio.3001551)
Supplement: S1 Table — (PDF) [file pbio.3001551.s014.pdf]

S1 Table. Inference of doubling times and effective population size [Ne] from direct estimates of cell numbers and nuclei per cells over the time course of two transfers.

Timepoint [h]	Cell numbers[1]	Nuclei (per cell)[1]	Nuclei (total)[2]	Nucleic doublings[3,4]	Population size [Ne][5]
0	1.65E+04	1.82	3.00E+04		
24	1.90E+04	9.69	1.84E+05	**2.62**	**7.86 E+04**
24	6.00E+02	9.69	5.81E+03		
48	6.00E+05	14.8	8.84E+06	**10.6**	**6.14E+04**

[1] determined experimentally

[2] calculated as product of *cell number* and *nuclei (per cell)*

[3] value at 24h: calculated as LOG2 (Nuclei,total) [24h] – LOG2 (Nuclei,total) [0h]

[4] value at 48h: calculated as LOG2 (Nuclei,total) [48h] – LOG2 (Nuclei,total) [24h]

[5] calculated as product of start (=bottleneck) cell numbers and nucleic doublings
