# Supplementary material for: Regulation of sedimentation rate shapes the evolution of multicellularity in a close unicellular relative of animals
Source: PLoS Biol. 2022 Mar 29;20(3):e3001551. doi: 10.1371/journal.pbio.3001551 (PMC8963540; doi:10.1371/journal.pbio.3001551)
Supplement: S2 Table — (PDF) [file pbio.3001551.s015.pdf]

S2 Table. Genome resequencing summary: data processing and mapping.

ID	Treatment (*)	Clumping Type (**)	Total Reads (***)	Raw Data (Gb)	Trimmed Reads (%) (****)	TrimmedData (Gb)	Aligned Data (%) (*****)	Deduplicated Data (%)	Informative Data (Gb)	Fraction of Reference Genome covered (%)	Effective Average Coverage
AN	A	A	64,961,014	8.12	44.6	4.50	99.31	96.74	4.35	88	34.5
S1	E	H	66,575,790	8.32	39.9	5.00	99.15	97.28	4.86	87	38.9
S2	E	I	60,999,004	7.62	40.5	4.54	99.25	97.46	4.42	87	35.4
S3	E	I	59,917,632	7.49	39.6	4.52	99.14	97.14	4.39	87	35.1
S4	E	H	57,928,616	7.24	40.5	4.31	99.24	97.35	4.19	87	33.8
S5	E	I	57,822,932	7.23	41.7	4.21	99.28	97.42	4.10	87	32.9
S6	E	L	57,657,624	7.21	40.0	4.32	99.29	97.50	4.22	88	33.6
S7	E	I	52,765,444	6.60	39.2	4.01	99.27	96.97	3.89	88	30.7
S8	E	L	52,155,682	6.52	42.2	3.77	99.35	97.16	3.66	87	29.3
S9	E	H	59,011,232	7.38	39.9	4.43	99.24	97.24	4.31	89	33.9
S10	E	L	57,388,476	7.17	40.1	4.30	99.47	96.84	4.16	90	32.2
Total			58,834,859	7.35	40.8	4.36	99.27	97.19	4.23	88	33.7
Control:											
DRR183670	R	C	116,162,364	29.0	45.4	15.9	98.37	96.63	15.3	94	114

(*) A, ancestral; E, evolved; R, reference
(**) A, ancestral; H, highly clumpy; I, Intermediate clumpiness; L, Low clumpiness; C, control
(***) Uniform raw read length of 125bp
(****) Data filtered during trimming prior to mapping
(*****) Given a reference genome size of 142,721,209bp
