# Supplementary material for: Regulation of sedimentation rate shapes the evolution of multicellularity in a close unicellular relative of animals
Source: PLoS Biol. 2022 Mar 29;20(3):e3001551. doi: 10.1371/journal.pbio.3001551 (PMC8963540; doi:10.1371/journal.pbio.3001551)
Supplement: S3 Table — (PDF) [file pbio.3001551.s016.pdf]

S3 Table. Summary and classification of annotated repeat regions in the *Sphaeroforma arctica* reference genome.

Name and classification of element	Number of elements	Length occupied [bp]	Percentage of total sequence[1] [%]
**Retroelements**	**44,831**	**5,606,241**	**3.99**
SINEs:	302	24,043	0.02
Penelope	3,232	256,219	0.18
LINEs:	29,324	3,997,048	2.84
CRE/SLACS	22	1078	0
L2/CR1/Rex	22,777	3,502,253	2.49
R1/LOA/Jockey	375	27,235	0.02
R2/R4/NeSL	126	8,005	0.01
RTE/Bov-B	479	38,312	0.03
L1/CIN4	1,779	117,738	0.08
LTR elements:	15,205	1,585,150	1.13
BEL/Pao	469	32,962	0.02
Ty1/Copia	651	145,765	0.1
Gypsy/DIRS1	7,466	999,654	0.71
Retroviral	5,423	322,159	0.23
**DNA transposons**	**89,667**	**8,369,254**	**5.95**
hobo-Activator	8,125	472,782	0.34
Tc1-IS630-Pogo	12,356	1,604,248	1.14
En-Spm	0	0	0
MuDR-IS905	0	0	0
PiggyBac	125	6,753	0
Tourist/Harbinger	1,314	67,309	0.05
Other: Mirage, P-element, Transib	2,894	195,557	0.14
**Rolling-circles**	**4,725**	**574,140**	**0.41**
**Unclassified**	**389**	**51,677**	**0.04**
**Total interspersed repeats**	**-**	**14,027,172**	**9.97**
**Small RNAs**	**1,146**	**1,193,846**	**0.85**
**Satellites**	**1,861**	**345,389**	**0.25**
**Simple repeats**	**73,308**	**12,722,055**	**9.05**
**Low complexity**	**2,982**	**166,665**	**0.12**

[1] Total reference genome size (Sarc4) is 142,721,209 bp distributed across 1,750 contigs with a GC-content of ~38.5%.
Masking of the entire portion (~20.6%) of annotated repetitive sequence resulted in a final repeat-masked genome length of 113,693,024 bp.
Beware that the lengths and percentage of total sequence do not directly add up to yield the above numbers, since repetitive regions may house overlapping annotations for different elements (e.g., simple repeats within other elements).
