# Supplementary material for: Regulation of sedimentation rate shapes the evolution of multicellularity in a close unicellular relative of animals
Source: PLoS Biol. 2022 Mar 29;20(3):e3001551. doi: 10.1371/journal.pbio.3001551 (PMC8963540; doi:10.1371/journal.pbio.3001551)
Supplement: S4 Table — (PDF) [file pbio.3001551.s017.pdf]

S4 Table. Variant calling.

								FAST			MEDIUM				SLOW			
ID	Seq id	Position	TYPE	Effect	Mutation	Annotation[1]	Affected genes	**S01**	**S04**	**S09**	**S02**	**S03**	**S05**	**S07**	**S06**	**S08**	**S10**	Read support[2]
1	scaffold658	3,101	SNP	NS	C→T	R90* (**C**GA→**T**GA)	Sarc4_g12019T	**1**										17/17
2	scaffold052	23,219	SNP	NS	A→G	E90G (G**A**A→G**G**A)	Sarc4_g23215T		**1**									25/25
3	scaffold209	85,868	SNP	NC	G→C	intronic **(-314/+740)**	Sarc4_g32431T		**1**									29/29
4	scaffold224	61,451	SNP	NS	T→A	S102C (**A**GC→**T**GC)	Sarc4_g33124T		**1**									12/12
5	scaffold226	196,494	SNP	IN	G→A	intergenic **(-587**/+1084)	Sarc4_g33270T		**1**									27/27
6	scaffold617	26,290	SNP	NC	C→T	intronic **(+390/-279)**	Sarc4_g11520T		**1**									30/30
7	scaffold042	318,312	SNP	NC	C→T	intronic **(+102/-711)**	Sarc4_g22580T			**1**								32/32
8	scaffold278	57,886	SNP	NC	A→G	intronic **(+291/-424)**	Sarc4_g3019T			**1**								33/33
9	scaffold426	46,771	SNP	NC	A→G	intronic **(+280/-543)**	Sarc4_g7653T			**1**								21/21
10	scaffold645	9,116	SNP	NS	C→T	A923V (G**C**A→G**T**A)	Sarc4_g11880T			**1**								22/22
11	scaffold651	57,503	SNP	NC	A→T	intronic **(+186/+1338)**	Sarc4_g11957 / Sarc4_g11958			**1**								30/30
12	scaffold241	79,469	SNP	IN	G→A	intergenic **(-268**/+232)	Sarc4_g33887T				**1**							23/23
13	scaffold406	53,611	SNP	NS	C→G	V176L (**G**TT→**C**TT)	Sarc4_g7170T				**1**							29/29
14	scaffold167	184,318	INS	FS	+AG	coding (**85**/117 nt)	Sarc4_g30293T					**1**						12/12
15	scaffold381	83,481	SNP	NS	C→T	G148S (**G**GT→**A**GT)	Sarc4_g6394T					**1**						24/26
16	scaffold153	151,457	SNP	IN	G→T	intergenic (+512**/-553**)	Sarc4_g29511T						**1**					35/35
17	scaffold414	97,263	SNP	NS	C→T	Q1750* (**C**AG→**T**AG)	Sarc4_g7365T						**1**					23/23
18	scaffold731	5,386	SNP	IN	A→G	intergenic (–/+118)	Sarc4_g12953						**1**					28/28
19	scaffold301	38,194	SNP	NS	C→T	D25N (**G**AC→**A**AC)	Sarc4_g3900T	**1**						**1**				37/37; 27/27
20	scaffold193	291,552	INS	IN	+G	intergenic (+3610**/-4749**)	Sarc4_g31776T								**1**			27/27
21	scaffold209	35,723	SNP	NC	C→T	intronic **(+360/-796)**	Sarc4_g32417T								**1**			28/28
22	scaffold336	12,377	SNP	NC	G→A	intronic **(+3872/-76)**	Sarc4_g4950T								**1**			25/25
23	scaffold842	3,222	SNP	IN	C→T	intergenic (**–**/+1674)	Sarc4_g14312									**1**		18/21
24	scaffold001	360,117	SNP	SY	T→C	P45P (CC**T**→CC**C**)	Sarc4_g18855										**1**	18/18
25	scaffold207	143,138	SNP	NS	G→A	R284C (**C**GC→**T**GC)	Sarc4_g32374T										**1**	17/17

SNP, single nucleotide polymorphism; IN, Insertion; NS, non-synonymous (including non-sense) mutations; SY, synonymous mutations; NC, non-coding (i.e., intronic) mutations; IN, intergenic mutations; FS, frame-shift mutation

[1] according to breseq's gdtools ANNOTATE (**bold**: changed base in codon (NS); position down/upstream of previous/next exon (NC); position down/upstream of previous/next gene (IN); position of insertion in coding sequence (FS))

[2] required variant allele support: $x \geq 10$, required majority frequency ≥ 0.8.
