# Supplementary material for: Regulation of sedimentation rate shapes the evolution of multicellularity in a close unicellular relative of animals
Source: PLoS Biol. 2022 Mar 29;20(3):e3001551. doi: 10.1371/journal.pbio.3001551 (PMC8963540; doi:10.1371/journal.pbio.3001551)
Supplement: S5 Table — (PDF) [file pbio.3001551.s018.pdf]

S5 Table. Transcript abundance of affected genes during *S. arctica* native life-cycle

ID	Affected genes	TYPE	Effect	Transcript abundance (in tpm)(1)									
				12h	18h	24h	30h	36h	42h	48h	54h	60h	66h
1	Sarc4_g12019T	SNP	SY	34.3687	37.1965	35.0699	37.0085	44.8557	97.1095	98.9791	86.1972	83.1505	61.6903
2	Sarc4_g23215T	SNP	NS	19.6851	19.1455	17.5439	16.2834	23.1461	18.6644	17.9979	19.4054	17.2579	11.882
3	Sarc4_g32431T	SNP	NC	69.5583	76.5122	76.5697	74.6316	91.2736	100.101	106.021	124.43	161.934	160.266
4	Sarc4_g33124T	SNP	NS	0	0	0	0	0	0	0	0	0	0
5	Sarc4_g33270T	SNP	IN	22.9663	28.5196	22.2241	23.1924	27.1365	21.835	19.1085	15.252	13.0743	11.0699
6	Sarc4_g11520T	SNP	NC	79.2703	83.0262	83.5683	75.0504	86.8678	124.211	171.462	169.185	127.728	72.5625
7	Sarc4_g22580T	SNP	NC	1.29071	1.55378	0.82560	0.84584	0.8672	1.17345	1.70523	2.04122	3.36911	4.0704
8	Sarc4_g3019T	SNP	NC	114.017	108.556	110.86	101.407	122.306	120.344	116.719	110.999	101.797	88.9507
9	Sarc4_g7653T	SNP	NC	16.3923	17.5438	16.7544	18.797	20.5921	19.6488	22.9749	23.9413	19.1717	15.4509
10	Sarc4_g11880T	SNP	NS	6.91487	6.60516	4.32566	4.51767	6.66984	4.26517	3.17804	2.43333	1.54733	2.50654
11	Sarc4_g11957	SNP	IN	0.55330	0.55777	0	0	0.33447	0.39681	0.84829	0	0	0
	Sarc4_g11958			0	0	0	0	0	0	0	0	0	0
12	Sarc4_g33887T	SNP	IN	0.25507	1.01635	0	0	0	0	0	0	0	0
13	Sarc4_g7170T	SNP	NS	0.31083	0.10386	0.11287	0.21621	0	0.37790	0	0.12197	0	0.28910
14	Sarc4_g30293T	INS	FS	23.7182	29.8256	31.1292	41.2785	57.8007	64.8248	71.5185	70.6809	60.6227	54.8389
15	Sarc4_g6394T	SNP	NS	19.3595	22.3913	19.4656	14.576	17.1674	28.6616	43.6442	131.04	64.7505	18.36
16	Sarc4_g29511T	SNP	IN	0	0.14618	0	0	0.15940	0	0.49779	0.17462	0	0.17259
17	Sarc4_g7365T	SNP	NS	41.1783	31.772	22.8192	27.044	34.9059	28.5977	19.6541	11.4106	12.3447	32.656
18	Sarc4_g12953	SNP	NC	57.216	69.811	67.439	66.957	97.928	115.4	118.91	129.18	142.42	131.73
19	Sarc4_g3900T	SNP	NS	0	0	0	0	0	0	0	0	0	0
20	Sarc4_g31776T	INS	IN	0	0	0	0	0	0	0	0	0	0
21	Sarc4_g32417T	SNP	NC	5.6145	6.5909	6.5687	7.5284	10.521	37.328	55.623	71.687	57.300	77.678
22	Sarc4_g4950T	SNP	NC	2.9573	2.0139	1.1597	2.5930	2.1831	7.3580	9.1223	12.325	18.977	22.469
23	Sarc4_g14312	SNP	IN	0	0	0	0	0	0	0	0	0.1011	0.1176
24	Sarc4_g18855	SNP	SY	14.359	38.709	33.958	49.115	51.133	63.816	65.745	82.792	92.719	71.047
25	Sarc4_g32374T	SNP	NS	0	0.0286	0	0	0	0	0	0	0	0

Reference

1. Dudin O, Ondracka A, Grau-Bové X, Haraldsen AA, Toyoda A, Suga H, et al. Erratum: Correction: A unicellular relative of animals generates a layer of polarized cells by actomyosin-dependent cellularization (eLife (2019) 8 PII: e60055). eLife. 2020.
