# Supplementary material for: Regulation of sedimentation rate shapes the evolution of multicellularity in a close unicellular relative of animals
Source: PLoS Biol. 2022 Mar 29;20(3):e3001551. doi: 10.1371/journal.pbio.3001551 (PMC8963540; doi:10.1371/journal.pbio.3001551)
Supplement: S6 Table — (PDF) [file pbio.3001551.s019.pdf]

S6 Table. Annotation and gene prediction of mutational targets.

ID	Locus tag	**Gene name**(1)	**Annotation**(1)	**COG assignment**(1)
1	Sarc4_g12019T	Ist1	Regulator of Vps4 activity in the multivesicular bodies (MVB) pathway	-
2	Sarc4_g23215T	SLC4A1AP, Kanadaptin	Solute carrier family 4 (Anion exchanger), member 1, adaptor protein	Signal Transduction (T)
3	Sarc4_g32431T	SLK	ste20-like kinase adaptor	Signal Transduction (T)
4	Sarc4_g33124T	-	-	-
5	Sarc4_g33270T	-	-	Function Unknown (S)
6	Sarc4_g11520T	DHCR7	delta(14)-sterol reductase, ERG4_ERG24	Lipid metabolism (I)
7	Sarc4_g22580T	DUF563	Domain of unknown function DUF563	-
8	Sarc4_g3019T	RCC1	Regulator of chromosome condensation (RCC1) repeat	Post-translational modification, protein turnover, chaperone functions (O)
9	Sarc4_g7653T	HLH	Helix-loop-helix DNA-binding, PHD finger superfamily; cl22851	-
10	Sarc4_g11880T	C2H2	Zn finger nucleotide-binding	-
11	Sarc4_g11957T / Sarc4_g11958T	-	-	-
12	Sarc4_g33887T	-	-	-
13	Sarc4_g7170T	-	-	-
14	Sarc4_g30293T	DAO	D-amino-acid oxidase	Intracellular trafficking and secretion (U)
15	Sarc4_g6394T	Erv26	Transmembrane adaptor	Lipid metabolism (I), Signal Transduction (T)
16	Sarc4_g29511T	-	-	-
17	Sarc4_g7365T	Fibrillin-2	Lectin C-type domain, EGF repeat-like domain	Signal Transduction (T)
18	Sarc4_g12953T	DNM1	Dynamin-related protein	Intracellular trafficking and secretion (U)
19	Sarc4_g3900T	-	-	-
20	Sarc4_g31776T	-	-	-
21	Sarc4_g32417T	IRK	Potassium inwardly-rectifying k+ (IRK) channel subfamily J, member (KCNJ6)	Inorganic ion transport and metabolism (P)
22	Sarc4_g4950T	-	-	-
23	Sarc4_g14312T	-	-	-
24	Sarc4_g18855T	RILP-like	Rab interacting lysosomal protein-like 1 and 2	Function Unknown (S)
25	Sarc4_g32374T	RVT_2	Retrotransposon protein	Function Unknown (S)

Reference

1. Grau-Bové X, Torruella G, Donachie S, Suga H, Leonard G, Richards TA, et al. Dynamics of genomic innovation in the unicellular ancestry of animals. Elife. 2017;
